# Challenges in Implementing Low-Latency Holographic-Type Communication Systems

**DOI:** 10.3390/s22249617

**Published:** 2022-12-08

**Authors:** Radostina Petkova, Vladimir Poulkov, Agata Manolova, Krasimir Tonchev

**Affiliations:** Faculty of Telecommunications, Technical University of Sofia, 8 Kliment Ohridski blvd., 1000 Sofia, Bulgaria

**Keywords:** HTC, HTC implementation challenges, HTC system

## Abstract

Holographic-type communication (HTC) permits new levels of engagement between remote users. It is anticipated that it will give a very immersive experience while enhancing the sense of spatial co-presence. In addition to the newly revealed advantages, however, stringent system requirements are imposed, such as multi-sensory and multi-dimensional data capture and reproduction, ultra-lightweight processing, ultra-low-latency transmission, realistic avatar embodiment conveying gestures and facial expressions, support for an arbitrary number of participants, etc. In this paper, we review the current limitations to the HTC system implementation and systemize the main challenges into a few major groups. Furthermore, we propose a conceptual framework for the realization of an HTC system that will guarantee the desired low-latency transmission, lightweight processing, and ease of scalability, all accompanied with a higher level of realism in human body appearance and dynamics.

## 1. Introduction

In the past ten years, there has been a lot of research in the field of holographic-type communication (HTC), including new ways of digital human representation and interaction. Technology advancements, particularly in three-dimensional (3D) data capturing techniques, processing power, virtual, augmented, and mixed reality (VR, AR, and MR) technologies and enabled devices, as well as networks of next generation, will soon make possible what has only been pictured in movies. The COVID-19 pandemic also gave a very big boost to the industry involvement in the development of holographic communication because billions of employees had to stay isolated for long periods of time with limited social interaction. The great benefits of HTC are the ability for immersive and spatially aware communication and interaction within six degrees of freedom between remote users, virtually represented in 3D space. Traditional two-dimensional (2D) video conferencing systems such as Webex, Zoom, MSTeams, etc., do not support the above and fail in providing consumers with the natural non-verbal cues that typically exists in real face-to-face communication scenarios such as gaze direction, gestures, tactile feedback, spatial faithfulness, depth perception of the physical environment, physiological signals, etc. [1]. This severely limits both the feeling of co-presence and the possibility for remote task collaboration. Many office workers will need more immersive kinds of digital interaction because the amount of time spent working outside the workplace is predicted to rise over the next ten years. When working remotely, more than half of them said they would like a multi-sensory digital workstation at the office [2]. On the other side, HTC appears as a promising solution that can even offer its users the benefit of the development of haptic technology, which adds the tactile sensing of digital items. Therefore, in addition to the basic communication purposes, it is justifiably considered in various applications, such as entertainment and gaming, architecture and construction, healthcare, education and training, tourism and heritage, industrial engineering, etc. [1,3,4].

An emblematic HTC system, called Holoportation, was developed by the Microsoft team in the middle of the past decade [5]. It is described as an end-to-end immersive system which is capable of real-time 360° capturing and transmission of people, objects, and movements. Each user who enters the captured space is virtually teleported to a remote site, where another user wearing an AR/VR headset can perceive him/her visually and audibly. Despite the significant breakthrough, the work’s great contributions were accompanied by some serious limitations, such as great amount of high-end hardware required for the functioning of the system, the need of high-capacity network connections allowing low-latency communication between the remote participants, the need for efficient compression schemes for lower bandwidth, the need for effective algorithms to correct the appearance of reconstruction artifacts, the need for real-time tracking algorithms, and development of algorithms for realistic real-time gaze-aware facial reenactment [5]. Moreover, one of the drawbacks was that system scalability was not considered at all. Even considering the latest developments in HTC, it should be noted that these limitations are still relevant.

The beneficial employment of HTC in various fields of applications requires thorough analysis on the experienced problems. Therefore, we aim to emphasize the key HTC system challenges and aid the research community and industry in the development of this immersive technology. Accordingly, we examine the latest works on the topic and systematize the most important challenges in implementation faced by any HTC systems. We also review a number of already existing HTC systems, comparing their performance parameters and inferring the challenges which they experience. Additionally, we believe that the path to the realization of holographic communication does not only go through technology advancements but also through applying smart ways of data processing, compression, visualization, and transmission. In this sense, we propose a conceptual framework for a Realistic Adaptive Dynamics Integration (RADI) system to enable an immersive, lightweight, and low-latency holographic type of communication. The contributions of this work are two-fold:

- Systematized review of HTC system implementation challenges and comparison between already implemented HTC systems;
- Development of a conceptual framework for a smart HTC system.

### 1.1. Literature Review and Selection Methodology

**Figure 1:**
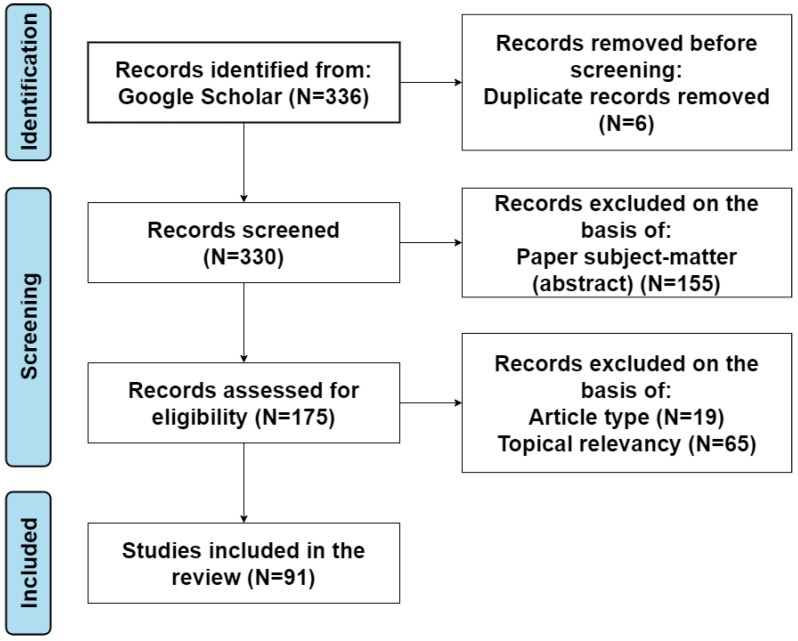
Summary of PRISMA flowchart of the article selection process for this review.

The review of existing HTC implementations is made by examining relevant papers on the topic from the past five years. A total of 91 papers out of 336 were chosen to be included in the review, after analyzing their contents and topics related to the concept of this work. The literature review was conducted following the Preferred Reporting Items for Systematic Reviews and Meta-Analyses statement (PRISMA) [6] and is illustrated in Figure 1.

We searched the Google Scholar database for papers relevant to the topic of HTC. The key words and word combinations which we used during the screening process were: holographic-type communication, holographic-type communication systems and challenges, ultra-low-latency communication, telepresence, MR telepresence systems, MR remote collaboration, AR/VR/MR communication, metaverse, immersive technologies, holographic technology, holographic displays, 3D displays, and others. The search resulted in 336 records, of which 6 were excluded due to record duplication; thus, 330 records were screened in total. Then, 155 more were excluded on the basis of the papers’ subject matter according to their abstracts. We assessed the remaining 175 papers as eligible for this study and examined their content in detail. Finally, 91 papers were considered as the most relevant on the topic and were included in the review of HTC system challenges.

### 1.2. Paper Structure

**Figure 2:**
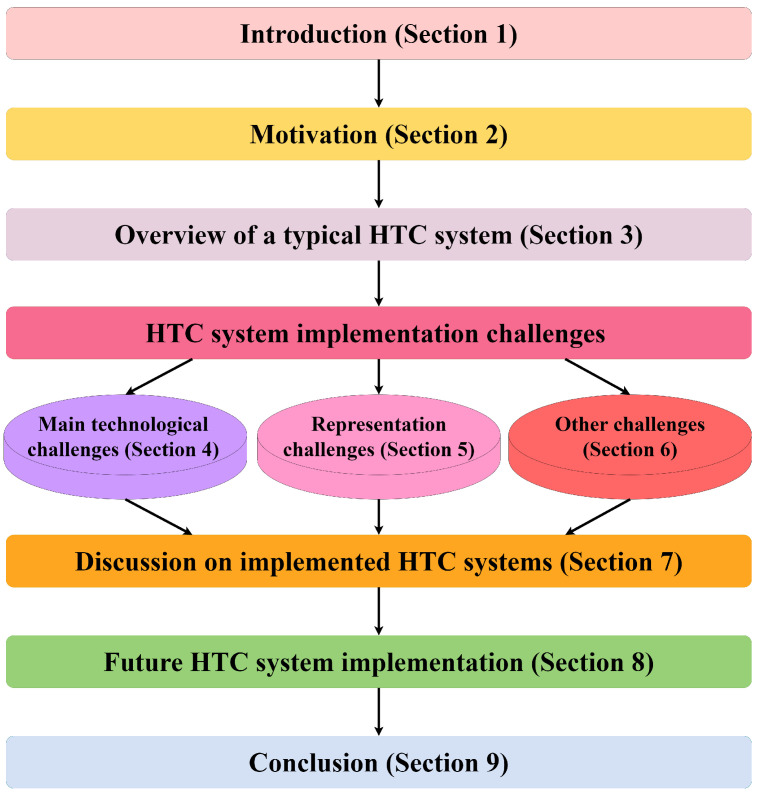
Paper structure.

The structure of the paper is illustrated in Figure 2. Section 1 contains a brief introduction to the topic. In Section 2, the motivation for this work is given. Section 3 presents an overview of the typical HTC system and gives an insight to the main processes that it should perform. HTC system challenges are summarized into three sections (Section 4, Section 5 and Section 6), each of which is subdivided into more subsections depending on specific elements. A discussion of the implemented HTC systems included in this review is presented in Section 7. Section 8 presents the proposed conceptual framework for a Realistic Adaptive Dynamics Integration (RADI) system. Finally, the paper is concluded in Section 9.

## 2. Motivation

Since HTC became an emerging topic in various fields of applications, a thorough analysis of its current realization limitations should be presented. To our best knowledge, by the time of writing this paper, no especially devoted works or holistic overviews of the HTC system’s implementation limitations and challenges exist. Most of the survey papers which we examined [1,3,4,7,8,9,10,11,12,13,14,15] focus on the current state of MR remote collaboration systems. These are systems that allow remote users to communicate, interact, and collaborate on different work tasks with the aid of the immersive AR and VR technologies without any geographic restrictions. However, the same survey papers do not extensively discuss the problems that such systems experience. Other works, such as [16,17], put an emphasis on challenges, but they do not put an emphasis on the problems that typical HTC systems experience. Table 1 presents an overview of the reviewed survey papers, with a comparison related to some performance parameters.

However, in order to fill the gap between the HTC system conceptualization and the HTC system realization for mass use, a complete knowledge of system challenges must be provided. The motivation behind this work is to clearly define the main HTC system implementation challenges and to aid researchers in the development of this prospective and exciting technology. In order to do this, we review above 30 works that describe the implementation of HTC systms and examine the challenges they face. To achieve a better overview of the experienced system limitations, we examine some other studies of HTC components specifics, such as data capturing [18,19,20,21,22], processing and transmission [23,24,25,26,27,28,29,30,31,32,33,34,35,36,37,38,39], visualization [12,40,41,42,43,44,45], system scalability [16,46], avatar embodiment [47,48,49,50], non-verbal cues [51,52,53,54,55], security and privacy [56,57,58], and system evaluation [59,60,61,62,63].

**Table 1:** Related surveys.

Reference	Year	Topic	Main Research Aspects
[3]	2019	Current state of collaborative MR technologies	A review of MR collaborative technologies according to their application area, types of display devices, collaboration setup, and user interaction and experience aspects.
[8]	2019	Revisiting collaboration through MR	A review of MR collaboration according to time and space dimensions, participants’ role symmetry, artificiality, collaboration focus, and collaboration scenario. Review of foreseeable direction for improving collaborative experience.
[9]	2019	A survey of AR/MR-based co-design in manufacturing	A review of state-of-the-art AR/MR-based co-designed systems in manufacturing in terms of remote and co-located collaboration, real-time multi-user collaboration, and independent view collaboration. Reviewing research challenges and future trends.
[13]	2020	A survey of collaborative work in AR	A review of papers along the dimensions of space, time, role symmetry, technology symmetry, and input and output modalities.
[14]	2020	A review of extended reality (XR) in spatial sciences	Review of research challenges and future directions in XR domain in terms of technology (display devices, tracking, control devices and paradigms for 3D user interface, visual realism, level of detail and graphic processing, bottlenecks in reconstruction and role of artificial intelligence (AI) in automation), design (visualization and interaction design), and human factors.
[15]	2020	A review of MR	A review following MR application framework that comprises a layer for main system components, a layer for architectural issues for component integration, an application layer, and a user interface layer.
[1]	2021	A review of AR/MR remote collaboration on physical tasks	A review of AR/MR remote collaboration papers from the following aspects: collection and classification research, using 3D scene reconstruction environment and live panorama, periodicals and conducting research, local and remote user interfaces, features of user interfaces, architecture and shared non-verbal cues, application, and toolkits. Review of limitations and open research issues in the field.
[4]	2021	A review of the existing methods and systems for remote collaboration	A review of Microsoft Hololens applications divided by application area, visualization technology (AR and MR), and functionality (visualization, interaction, and immersion).
[7]	2021	A survey of synchronous AR, VR, and MR systems for remote collaboration	A review of the recent developments of synchronous remote collaboration systems in terms of environment (meeting, design, and remote expert), avatars (cartoon, realistic, full body, head and hands, upper body, reconstructed model, video, AR annotations, hands only, and audio avatar), and interaction (media sharing, shared 3D object manipulation, 2D drawing, mid-air 3D drawing, AR annotations, AR viewpoint sharing, hand and gestures, shared gaze awareness, and conveying facial expression).
[10]	2021	A survey of spatially faithful telepresence	A review of the status of natural perception support, mobility and viewpoints serving, transmitting and displaying volumetric videos, viewpoint-on-demand, photo realism vs virtual world approaches, glass-based vs screen displays, and XR’s role in 3D telepresence.
[11]	2021	A review of the existing methods and systems for remote collaboration	A review of the following remote collaboration aspects: electronic devices (video cameras, projectors, display devices, HMD, smart devices, and robot devices); displaying technologies (2D video stream, 3D view, 360° panorama, AR, VR, and MR); and communication cues (annotations, pointing, gestures, and gaze).
[12]	2021	A review of the current state of holography and 3D displays	A review of recent accomplishments made in the field of holographic 3D displays, holographic data transmission, and rendering hardware.
[17]	2021	“Telelife” and the challenges of remote living	Research on the “Telelife” challenges of remote interfaces, smart homes, learning, collaborating, privacy, security, accessibility, adoption, and ethics. Reviewing the grand technical challenges facing the “Telelife” implementation.
[16]	2022	Scalable XR in the dimensions of collaboration, visualization, and interaction	A review of scalable XR according to its collaboration support features, consistent and accessible visualization, and intuitive interaction techniques. Review of future research directions in the face of general research topics, scalability between different devices, scalability between different degrees of virtuality, and scalability between different number of collaborators.
**THIS PAPER**		**A review of HTC system implementation challenges**	**A review of the challenges to the HTC system implementation systematized in the following three groups: main technological challenges (input and output (I/O) challenges, data processing challenges, data transmission challenges, and HTC system scalability challenges), representation challenges (avatar embodiment challenges, gesture support challenges, gaze support challenges, and emotions support challenges), and other challenges (HTC system evaluation challenges and security and privacy challenges). Comparison between already existing HTC systems. Proposal of a conceptual framework for future HTC system implementation.**

## 3. Overview of a Basic HTC System

**Figure 3:**
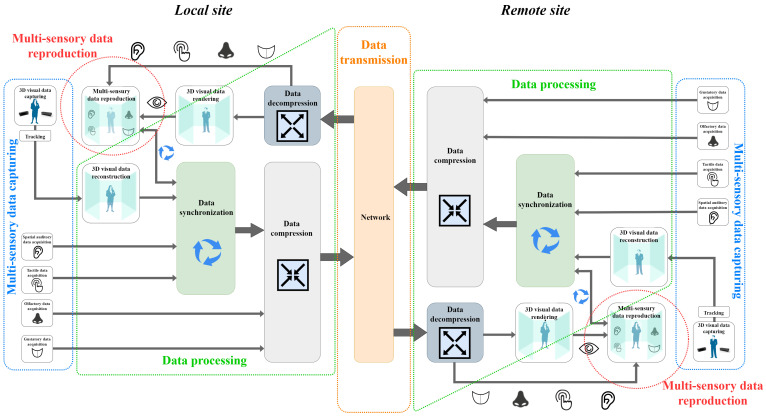
HTC system overview.

In general, to implement a low-latency HTC system, there are a few main operations that must be performed at both local and remote user sites. These are multi-sensory data capturing and reproduction, data processing, and data transmission. A block diagram of a basic HTC system is visualized in Figure 3. When a user acts as a transmitter in the forward direction of the holographic communication pipeline, he/she (and eventually his/her local space) must be faithfully captured, processed, and transmitted to the remote interlocutor. The capturing step involves spatially aware data acquisition for all the five human senses, i.e., visual, auditory, tactile, olfactory, and gustatory, performed by the blocks of 3D data capturing, spacial auditory data acquisition, tactile data acquisition, olfactory data acquisition, and gustatory data acquisition, respectively. If multiple visual data sources are used, they must be preliminarily synchronized. The tracking block is involved in the capturing step to dynamically track the user’s gestures and movements. Then, the acquired data are subjected to various processing measures, such as 3D visual data reconstruction, multi-source stream synchronization, and data compression. The 3D data reconstruction is performed by the block of 3D data reconstruction, where multi-view data alignment, noise filtering, hole filling, mesh fitting, object detection and extraction, etc., are performed. The synchronization between the streams coming from the local site, and the synchronization between the local and remote signals are both completed by the data synchronization block. The data compression is performed by the data compression block. Finally, the heterogeneous data are transmitted through a network channel, as the channel is represented by the network block. When the same user acts as a receiver in the backward direction of the holographic communication pipeline, he/she needs to receive, process, and reproduce the data obtained from the remote interlocutor. First, the received data are decompressed by the data decompression block. Then, their visual part is spatially rendered by the 3D data rendering block, and together with the remaining multi-sensory information, they are synchronously reproduced to the local user via the multi-sensory data reproduction block. Note that the forward and backward direction performances are carried out simultaneously.

One typical application of such an HTC system is teleconsultation, where a local expert may be assisted by a remote specialist during specific task completion. Teleconsultation can be useful in various fields, such as medicine, architecture, education, design, etc. For this purpose, the remote specialist has to be immersed in the local expert’s environment in real time. Therefore, the local expert and his/her surroundings must be captured, reconstructed, and further transmitted with low-latency to the remote specialist. Then, the remote specialist will be able to faithfully perceive the captured space and to immediately give instructions for the specific task completion. At the same time, the specialist can also be captured, reconstructed, and transmitted to the local expert, making him/her feel as if they are both present in the same space. The spatially aware communication between the participants and the possibility of conveying non-verbal cues such as gaze, gestures and face expressions permit new levels of remote user engagement. The interaction becomes much more realistic, comparable to a real face-to-face scenario, and enables faster and intuitive collaboration.

**Figure 4:**
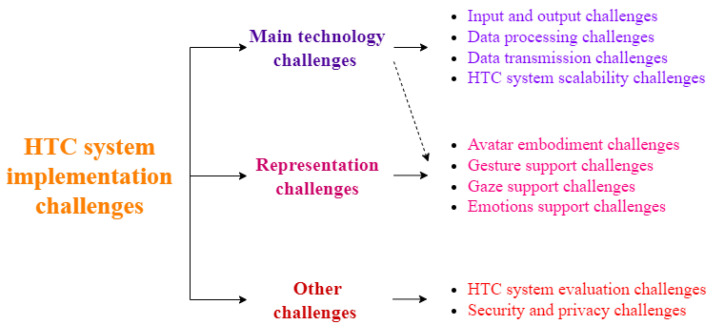
HTC system challenges classification.

However, despite recent advances in multi-sensory data capturing and reproduction, the increase in processing power, and the improvement in next-generation networks, it seems that technology is still not matured enough to fully support the holographic type of communication. In terms of current technology, the weaknesses in the major HTC system components mentioned in the previous paragraph are discussed in the following Section 4. User representation challenges (occurred due to some technology shortcomings), as well as other important problems, are discussed later in Section 5 and Section 6. The classification of the HTC system implementation challenges on which the paper flow is based is given in Figure 4.

## 4. Main Technological Challenges in Implementing HTC

### 4.1. I/O Technologies

Visual perception is usually considered as the most important of the human senses; therefore, I/O technology efforts are mainly concentrated on 3D visual content capturing and visualization. However, even though volumetric data are much more informative than 2D content, they are also more challenging to be obtained, especially in the context of HTC. This is due to several reasons. First, data capturing is required to be performed in real time. This automatically excludes the usage of highly accurate laser scanners or 3D reconstruction from multiple 2D images on a photogrammetry basis. Consequently, the second main challenge is the accuracy of the acquired data. The fulfillment of the real-time capturing requirement forces the developers to rely on more inaccurate but real-time-enabled devices, such as structured light-based sensors (PrimeSense cameras, Kinect for Xbox 360 sensor), Time-of-Flight (ToF) cameras (Microsoft Kinect v2 and Kinect Azure, [19,20,22]), or stereo cameras (Intel RealSense cameras D series, [18]). A comparison of the three methods for 3D data acquisition is given in [21]. Currently, because of their disadvantages, structured light sensors are sparsely used in contrast to the other two types of technologies. However, they also suffer from low precision capturing capabilities, low resolution, distance range limitations (for both long and short perspectives), and most importantly—noise addition. Another key limitation is the narrow field of view (FOV). As an example, the Kinect Azure reaches 120° × 120°, which is still much less than what human eyes may perceive (200–220° × 130–135°). The narrow FOV, as well as the fact that an object cannot be captured entirely from a single shot, impose the need of a multi-camera setup. This requires the deployment of more than one camera sensors that must be precisely calibrated. However, although camera calibration is a well-studied topic, it enforces the usage of calibration markers, can produce appearance of alignment errors, and has lower reconstruction precision. Multiple camera installation in a customer scenario is also a hurdle because of the significant increase in the financial costs. Using 360° cameras (e.g., Ricoh Theta, Samsung 360 Round, Insta 360 Pro, etc.) is also a popular solution to extensively capture the environment. They provide a high level of immersion in terms of visualization, but user movements are limited to the camera position only, which corresponds to perceiving just three degrees of freedom [64]. Finally, when speaking of capturing dynamics, high frame rates are required to optimally convey the kinematics of movements without any lagging. Naturally, this leads to increasing computational and transmission overload.

Along with 3D visual content capturing, 3D visual content visualization is an indispensable part of one HTC system. An ideal 3D displaying technology must provide users with highly immersive experiences supported by very high-resolution imaging within a large FOV and a possibility for real-time interaction [44]. These factors must be simultaneously maintained while ensuring the user perception with the following physical (depth) cues: accommodation, conversion, stereo disparity, and motion parallax. Currently, based on the reviewed papers on telepresence systems for remote communication and collaboration, VR/AR head-mounted displays (HMDs) are the most often used in terms of visualization. A comparison between different AR and VR headsets available on the market can be found in [45]. However, even though considered as the technology that can provide the highest immersive experience, HMDs are still far from reaching their full potential of mimicking the human visual system. The FOV and the resolution provided by the headsets are less than what human eyes may experience in real conditions, which impacts the level of the perceived Quality of Experience (QoE). Another challenge in designing 3D displays is the provision of user comfort [44]. In [40], some disadvantages of the current HMDs are indicated, such as limited battery life, limited usage by a single user at the same time, heaviness and inconvenience, obtrusiveness, low availability, high setup cost, and high requirements on computation hardware. However, the greatest obstacle to providing comfort is the so-called convergence–accommodation conflict. It is expressed in the mismatch of the lens accommodation and the eye convergence depth cues. The image that is projected on the HMD is displayed at a fixed distance from the human eye, which produces constant accommodation, while the convergence angle may vary according to the scene [12]. This is reflected in the user experiencing discomfort such as nausea, dizziness, oculomotor, and disorientation. Some other factors such as quality of visualization, visual simulation, type of content, type of user locomotion, and time of HMD exposure may also provoke the user to experience some type of sickness. The authors of [42] extensively surveyed the impact of the above factors in the occurrence of nausea, oculomotor, and disorientation experiences by some VR users. However, along with the technical and comfort problems, the challenge of using HMD in shared and social spaces also needs attention [43]. This includes the social acceptability of HMD, tracking isolation and exclusion, shared experiences in shared spaces, and ethical implications of public MR. To combat the limitations of HMD displays, super-multi-view displays or light field displays (LFD) [12,40,41] and volumetric displays [12] are starting to appear. They are considered as non-obtrusive systems that may be observed by an arbitrary number of users (with some considerations); the same is not possible with the HMD. LFDs fight the accommodation–convergence conflict by being able to reproduce some level of accommodation. This is possible thanks to moving the image plane in and out of the display plane by redirecting the light rays to different voxel regions of the display panel [12]. However, the continuous increase in the provided depth is limited by diffraction occurrence among the voxels [12]. Research efforts are directed toward multi-plane LFD, but occlusions between the different planes cause new types of difficulties [12]. Significant work on LFD technology is presented by Holgrafika [65]. In [12], volumetric displays are considered as being able to overcome some of the multi-plane LFD limitations, but again, the projection of an image outside the panel volume is not possible. The authors of [12] outline the three main challenges impeding holography: realistic holographic pattern computation from the 3D information in a reasonable amount of time, data transmission to the visualization technology, and development of a suitable 3D display that can reproduce large holograms with high resolution at high refresh rates.

Except for the visual perception, a real face-to-face communication scenario involves the utilization of all others human senses—auditory, tactile, olfactory, and gustatory. However, most of the studies concentrate their efforts just on visual perception, aiming to provide realistic spatial information for users and their environments. Meanwhile, they fail to address the need for spatial consistency between video and audio, which is a mandatory condition for the realism of audio–visual perception. Just a few of the examined studies consider the case of implementing spatial audio [66] or declare the need for it [17,67,68]. Similar to auditory sensation, tactility is also a premise for immersive interaction, but it is usually neglected due to its challenging integration in the communication systems [69]. Currently, the most common tactile appliances are the so-called smart gloves and e-skin-based interfaces, which are often uncomfortable to use and thus negatively impact users’ QoE. Another issue is how to ensure bidirectional interaction by simultaneously providing tactile sensation and localized haptic feedback, which is still in the infancy stage of research. However, many studies declare the need for ensuring haptic feedback [52,70] and multi-sensory interaction for all participants [17,49,68,71,72]. The stimulation of the last two human senses, namely smell and taste, is even more limited and much more challenging to incorporate in future HTC systems. Currently, SENSIKS [73] is an appropriate example of involvement of the so-called sensory reality pods. They are closed and controllable cabinets devoted to provide multi-sensory experiences equipped with different programmable actuators. Although it is a promising approach for providing immersive interaction including all five human senses, it is not applicable for commercialization in future HTC systems due to its high cost, especially when multi-user scenarios are involved. The challenges of I/O modalities for HTC systems are summarized in Table 2.

**Table 2:** I/O challenges.

Group of Challenges	Challenges
**Visual input**	Depth cameras: low precision, low resolution, distance range limitations, noise addition, and narrow FoV [21];
	Multi-camera set up: need of calibration, alignment errors, lower reconstruction precision, time expense, and hurdle installation;
	360° capturing: limited user movements [64];
	Computational and transmission overload for higher frame rates.
**Visual output**	HMDs: less resolution and narrower FoV than human eye visual system [45];
	Limited battery life, limited usage to a single user at a time, heaviness, inconvenience, invasiveness, low availability, high set up cost, and great requirements on computation hardware [40] ;
	Convergence–accommodation conflict, discomfort, nausea, dizziness, oculomotor, and disorientation [12,42,44];
	Social acceptability of HDM, tracking isolation and exclusion, shared experience and shared space, and ethical implications of public MR [43];
	LFD and volumetric displays: limited panel volume, limited depth cue, and great amount of data [12,40,41];
	Simultaneous provision of highly immersive experience supported by very high resolution within very large FoV and possibility of real-time interaction [44].
**Other types of I/O technologies**	Need for multi-sensory interactions [17,49,68,71,72];
	Need for video and audio consistency (need for spatial audio) [17,67,68];
	Integration of tactility in HTC systems and difficulties in providing bidirectional tactile sensation and haptic feedback [52,69,70];
	Limited incorporation of smell and taste and high cost of integration of sensory reality pods.

### 4.2. Data Processing

The main challenge to data processing in HTC systems is how to ensure the ultra-lightweight computation of great amounts of data with low latency. The processing may include various operations depending on the use case scenario. Here, only the compulsory ones are discussed. These are: 3D data reconstruction and rendering, compression and decompression, and stream synchronization.

The 3D reconstruction of visual data captured from multiple perspectives requires a precise camera calibration. However, even with perfect calibration, the alignment often results in visual artifacts. So, further processing such as noise filtering, hole filling, etc., is required, leading to additional time expense. However, the greatest obstacle is not that multiple tasks need to be performed but the big amount of data that must be processed. Imagine working with dense point-cloud data, this means that billions of 3D points must be processed. Additionally, if we take into account a mesh structure, then the connections between the 3D points (the vertices) should also be considered.

Such volumes of data are also challenging for channel transmission. Therefore, to reduce the transmission overload and to decrease the subsequent bandwidth requirements, optimized compression and decompression techniques are mandatory. Recently, the Moving Picture Experts Group (MPEG) developed two point-cloud compression (PCC) techniques, called geometry-based PCC and video-based PCC [31]. While the video-based PCC projects the 3D data over 2D plane images and further utilizes the existent image compression techniques, the geometry-based PCC works directly on the point-cloud data. Each of the methods has their advantages and appropriate field of application [28,30,31,32]. However, determining the spatial–temporal correlation of a dynamic point cloud is still a tremendous task, especially when near to real-time transmission is required. Currently, deep learning methods are attracting great attention in the context of density point-cloud compression [23,28,33,34,36]. However, encoding and decoding times still exceed the run-time limits that low-latency communication imposes, and further improvements are needed in this direction. To decrease the compression time, either the amount of data should be reduced, or the computation power should be increased [26]. Therefore, it is essential to find a trade-off between developing efficient compression techniques, thus increasing the computational latency while decreasing the network bandwidth and latency, and vice versa [27].

In terms of 3D data rendering (according to the data obtained from the remote user), the quality of rendered 3D objects is a function of the wireless channel quality, known as cliff and leveling effect. The authors of [23,25] propose upgraded point-cloud delivery schemes based on graph neural networks and Graph Fourier Transform, where, however, rendering quality improvements according to the wireless channel state require additional communication overload.

The stream synchronization, both between the signals originating from different sensors in the local site and between the streams sourcing from different sites, is one of the stringent HTC requirements. Speaking of multi-sensor stream synchronization means that all types of signals coming from the local site, e.g., video, audio, and tactile, must be accurately synchronized [27] to guarantee the faithfulness of users’ perception. Moreover, if the visual information is to be obtained by exploiting several capturing devices in a dynamic scenario, all the devices must be synchronized to ensure complete and consistent imaging and movement of the reconstructed objects. In a multi-user holographic communication setup, the synchronization of the streams sourcing from different participants sites is mandatory. A few factors may lead to an increase in synchronization errors, including network distance between the participant sites, resulting in experienced latency, varying network path conditions, and source frame production conditions [37]. The network latency evaluation is mandatory, but it is not enough to choose the synchronization approach. The changing network conditions could reveal varying latency times, known as jitter, which may strongly corrupt the synchronization of the arriving streams, further resulting in decreased QoE. In [39], a novel cloud-based HTC teleportation platform is proposed which supports adaptive frame buffering and end-to-end signaling techniques against the network uncertainties. In [37], the performance of an edge-computing-based mechanism for stream synchronization is evaluated under different network conditions. However, working on synchronization mechanisms and their evaluation within systems with a much greater number of users performing faster dynamics is a challenge to be considered as research on HTC progresses.

Considering all the operations above and other additional processing such as user/object detection, extraction, tracking, positioning, mesh reconstruction, etc., it is obvious that the ultra-lightweight processing is really a challenge. Powerful graphical processing units are very helpful but not always available at the user site. Therefore, using the advantage of the network edge computing would be necessary for customer support [26,38,39]. Data processing challenges are given in Table 3.

### 4.3. Data Transmission of Holographic Data

In this subsection, three main challenges related to holographic data transmission are considered: ultra-high bandwidth, ultra-low-latency, and network optimization [27]. Let us assume the following scenario: three Kinect v2 sensors capture a dynamic scene at 30 fps. Each sensor provides point-cloud data with 217,088 points per frame, which gives a total of 651,264 points per frame for the three sensors. For each single point, geometry characteristics are represented by 32-bit x, y, and z values, and color attributes are described with 8-bit r, g, and b values. The calculation for the total amount of data at 30 fps is 651,264 × (3 × 32 + 3 × 8) × 30 = 2,344,550,400 bps. That is approximately 2.2 Gbps. In addition, assuming light field visualization that demands super-multi-view capturing, this will result in a much greater amount of information, going as high as Tbps. As a result, significant bandwidth resources will be required. It is obvious that HTC imposes huge network throughput demands. The giga- and terabits per second rates require ultra-high transmission bandwidths and the utilization of higher frequency bands, in addition to the application of efficient modulation techniques. Although 5G promises to support such demands [74], the realization of HTC is in a development stage and still very far from true commercialization. To lighten the bandwidth requirement, one effective technique is adaptive streaming [75], benefiting from the knowledge of users’ location and focus. This includes the transmission of just the user’s observable parts of the scene [35], as well as the transmission of point-cloud objects that are closer to the user with higher density [46]. Thus, the bandwidth consumption can be significantly reduced while still perceiving the same QoE. This solution, however, requires semantic knowledge of the scene [26] and accurate user motion and gaze tracking. Applying user view-point prediction is beneficial for volumetric streaming in an efficient manner [26].

The second challenge is the achievement of end-to-end low-latency transmission—from the local site capturing to the remote site rendering. At each step, including data acquisition and reconstruction, application-specific processing, compression, transmission, decompression, rendering, and visualization, additional delay is imposed. All the reviewed studies that evaluate experienced latency declare values of a couple of hundreds of milliseconds [49,64,66,71,76,77,78,79,80,81], which is too much for HTC (fewer than 15 ms motion-to-photon latency is acceptable according to [82,83], and fewer than 50–100 ms end-to-end latency according to [83]). A devoted transmission scheme for ultra-low-latency HTC is strongly needed. Based on the literature review, the current networking technologies that are increasingly exploited are the real-time streaming protocol (RTSP) [49,77,79] and dynamic adaptive streaming over HTTP (DASH) [75,78,84]. Although not originally invented for HTC purposes, WebRTC platform [66,76,85,86,87] and Photon Unity Network (PUN) module [47,50,67,85,88,89,90] are commonly used by HTC system developers. However, to the best of our knowledge, a protocol that is specially designed for HTC does not exist. Usual transport layer protocols support either low-latency (user datagram protocol (UDP)-based) or reliability (transmission control protocol (TCP)-based), or exploit some mechanisms to try to combine both (e.g., quick UDP internet connections (QUIC)). Nonetheless, none of them are absolutely suitable for holographic data transmission, so an optimized solution is still in demand [26]. The above transmission challenges are summarized in Table 3.

The third challenge is the network structure optimization, which is to aid in the fulfillment of the increasing HTC requirements, particularly ultra-lightweight processing, ultra-low-latency, and ultra-high bandwidth. The authors of [91] declare the need for an intelligent approach for network organization, where AI will help the network to constantly adapt according to its resource availability and depending on the HTC users’ behavior. The authors also state that a significant part of the computation must be migrated from the user site to the network, so FoV and resolution can be increased, and interactions using natural gestures can be easily enabled. The migration of the computation to the network site can significantly reduce the energy consumption of XR devices, thus increasing their battery life. Therefore, a higher degree of functionality can be added while reducing devices’ size and weight and enhancing user comfort. However, such computing and network upgrades must be achieved without trade-offs in terms of cost and quality [91]. According to the International Telecommunication Union (ITU)’s technical report [92], AI has a main role in optimizing the performance of future networks (Intelligent Operation Networks (IONs), as the authors call them). IONs must support computing-aware network capabilities and intelligent load-balancing simultaneously among multiple users in a coordinated way. Therefore, they must be adjustable according to the services demanded and network resources available. The requirements of IONs are computing awareness (since network and computing converge), joint network and computing resource scheduling, network protocol programmability (flexible configuration of future networks’ resources), flexible addressing, distributed and intelligent network management, multiple access capability, and fast routing and re-routing. In [93], AI is again indicated as a key technology with which to support AI-based service awareness capabilities. According to the authors, networks have to provide individual users with efficient coding and decoding, optimized transmission, QoE assurance, and coordinated scheduling capabilities for full-sensing holographic communication services. The authors of [27] propose “Cross layer optimization approach”. It includes end-user optimizations (view-point prediction, sensor synchronization, Quality of Service (QoS)/QoE/cybersickness assessment, and 3D tiling and multiple representation encoding), transport layer optimizations (low-latency optimization, intelligent buffering, caching, and smarter re-transmission), and novel network architectures. According to the novel network architectures, different Software-Defined Network (SDN) architectures must be examined, so better network performance for HTC applications can to be achieved. A fully distributed SDN architecture is indicated by the authors to achieve the lowest latency. Additionally, Network Slicing and Service Function Chaining (SFC) are also considered to further optimize the use of network resources. To conclude, applying strategies for intelligent network deployment is very important for future holographic service enablement. First, it can play a significant role in the optimization of holographic data transportation via the intelligent utilization of network resources. Second, such a deployment can be very beneficial for the end users who are not able to perform the heavy computation demanded by HTC.

### 4.4. System Scalability

For a system to be considered as scalable, it must be able to accommodate and serve an arbitrary number of users. In current 2D video conferencing systems, this is not an issue, because they can support the communication between multiple users. However, when talking about scaling the HTC system, there are plenty of issues. The bandwidth requirements go higher, the low latency must be assured along a greater number of users, and the synchronization and fusion (including tracking and positioning) between the user avatars, movements, environments, etc., become even more of a complex task. Scalability between different devices, between a different number of collaborators, and between different degrees of virtuality should be also considered [16]. Solutions with centralized control such as the one described in [46] will be needed. Some of the reviewed papers consider their systems as scalable, however, they support only up to a few users and do not manage to implement fully immersive low-latency HTC [47,50,70,71,77,78,87,88,90,94,95,96,97,98]. The scalability challenges are summarized in Table 3.

**Table 3:** Data processing, transmission, and scalability challenges.

Group of Challenges	Challenges
**Data processing**	Processing of great amount of data, high-quality reconstruction (in multi-camera set up), and efficient compression/decompression techniques in a reasonable amount of time;
	Requirement of great computational power;
	Trade-off between compression latency and network latency [12,23,25,26,27,31];
	Advantaging network edge computing [26,38,39];
	Multi-sensor stream synchronization of local site signals [27];
	Synchronization of streams coming from multiple sensors [37,39].
**Transmission**	Ultra-high bandwidth, efficient modulation techniques, and higher frequency bands;
	Applying adaptive streaming [35,46];
	Semantic knowledge and view-point prediction [26];
	Low-latency transmission fewer than 15 ms motion-to-photon latency and fewer than 50–100 ms end-to-end latency [64,76,77,78,79,80,81,82,83];
	Development of an optimized mechanism for holographic data transmission [27];
	Network structure optimization [27,91,92,93].
**Scalability**	Increasing technology requirements—higher bandwidth, insurance of low latency, and synchronization across many users;
	Need for centralized control [46];
	Considering scalability between different devices, between a different number of collaborators, and between different degrees of virtuality [16].

## 5. Representation Challenges

Except for the main technology challenges, HTC implementation also experiences problems related to users’ 3D representation. This is why they are called representation challenges. These are avatar embodiment, support of gestures, eye gaze, and emotion expressions. They usually occur due to technology and application limitations.

### 5.1. Avatar Embodiment

Accomplishing distant “face-to-face” low-latency communication is the foundation of HTC. In terms of technology, the expression “face-to-face” is visualized through capturing and reconstructing users’ digital representations, called virtual avatars. In the sense of avatars being the virtual versions of the real humans, they need to properly address humans’ features, e.g., visual representation, facial expressions and emotions, body movements and gestures, eye gaze, and other non-verbal cues. However, the construction of an avatar in a human-like manner is still a challenging task due to the various aspects that need to be considered [51]—providing a high sense of presence and realism, high detail of the avatars’ faces and facial expressions, completeness of the avatars’ bodies and related positions, fidelity of movements and gestures, locomotion synchronization between the real users and their avatars, providing a high sense of body ownership and ability to recognize users’ self-movements, and the avatars’ placement and cooperation in the interaction space. According to [50], there are two main avatar aspects that need to be extensively analyzed—visual fidelity and kinematic fidelity. In [68], the authors also emphasize the achievement of realistic appearance and behavioral realism.

According to avatar construction, two main approaches of virtual human embodiment can be outlined. The first approach focuses on capturing the real shape of the user, and it may be defined as photo-realistic [10]. It relies on real-time data acquisition and reconstruction of a human body using devices such as the Kinect sensors, Intel Real Sense cameras, or others. The requirement of immediate capturing and avatar construction forces the avoidance of professional scanning devices due to their requirement for long-time data acquisition. Therefore, the result of the real-time capturing process is usually a noisy and incomplete representation of the user, which disrupts visual quality and imposes some additional processing as noise filtering and hole filling. However, high realism and quality of representation still come at the cost of some computation load, processing delay, and energy consumption. Considering the replication of the body’s locomotion and gestures, the photo-realistic approach ensures better behavioral realism, especially if the body can be fully captured and has space to move freely. In theory, this statement also applies for mimicking finer gestures and facial expression, but only if the same are captured in detail. In practice, this is almost impossible because of the precision limitations of the devoted devices, as well as face occlusions due to HMDs. Therefore, computer vision and AI methods are needed to restore the incomplete and missing data.

The second approach for avatar embodiment solves some of the above problems, performing the avatar construction before the communication process begins. This type of avatar usually does not correspond to the actual user’s representation, and the avatar is considered as predesigned [71] or character-based [49]. The two main benefits of this approach are very good visual quality at high resolution and the avoidance of heavy computer graphic processing in real time. However, although visual quality may be very high and optimal, and avatar customization (gender, ethnicity, height, body shape, eye color, hairstyle, emotions, and clothing) may be achieved at some level, it is quite questionable if this representation can provide the desired realistic appearance. Replicating real users’ movements is possible in two cases—first, if the real human body is tracked by cameras or equipped with some tracking devices attached to the head/face, hands, torso, and limbs, and second, if the predesigned avatars allow deformation and outside control. Attaching hardware to the body is often experienced as intrusive and uncomfortable by the users. Exploiting tracking cameras, on the other hand, fails in tracking finer gestures and facial expression. Meanwhile, for the predesigned avatars to be pliable to outside control, and especially to be highly deformable, is a very important condition to ensure the requirement of behavior realism. Even so, they still experience some unnaturalness in the way they move. Additionally, for ensuring visually plausible representation of the avatar, high-quality movements of the garments need to be simulated [24]. The deformation of garments and bodies is a tricky but mandatory task in order to reproduce the expected level of realism. It requires the intervention of computer vision, optimization tasks solving, and AI, which, for sure, implies additional computation and latency.

The authors of [49] pose the question of whether the predesigned high-quality avatars with higher degree of freedom in expression realism would better convey information and ensure higher levels of presence than realistic but noisy and incomplete point-cloud avatars. The result of their social experiment is precise, stating that the realistic point-cloud models are superior to the predesigned ones regarding perceived co-presence, social presence, behavioral impression, and humanness. The authors concluded that even with worse visual quality, especially in the facial area, realistic point-cloud avatars resulted in better conveyance of user behavior and in a more coherent fit into the simulation context.

In conclusion, most of the examined studies for real-time MR remote communication and collaboration that extensively discuss the application of human model embodiment claim the need for more realistic and human-like avatars in both terms—visual representation and behavior realism [24,47,49,50,54,66,67,68,77,96,99,100].

### 5.2. Gesture Support

Another important aspect for an immersive social experience is the ability of sharing different non-verbal communication cues, such as head and body position and movement, hand and body gestures, gaze direction, etc. This is not possible with traditional 2D video conference systems; thus, supporting feelings of trust, rapport, togetherness, empathy, and signals of intimacy is highly limited. However, linking all these non-verbal cues in a 3D telepresence is also challenging. First, obtaining accurate body position, capturing, and then mimicking movements and gestures requires dynamic tracking and rendering of the human body. For some special collaborative tasks, obtaining finer hand gestures is required. As already mentioned, this is often accompanied by uncomfortable tracking devices attached to the body (body suits, gloves, head trackers, finger tracking sensors such as Leap Motion, etc.), or by markers and additional computer processing in the case of camera tracking. Perfect calibration between the different users’ coordinate systems, as well as stringent time synchronization, are mandatory so that an optimal fusion between the users in the meeting environment may be achieved. However, real human users’ and virtual avatars’ synchronization might also be a problematic task. According to [67], the direct synchronization of the local user movements and gestures with his/her devoted avatar may result in inaccurate information conveyance at the remote user site due to the dissimilarity of the two physical environments including size, configuration, and spatial arrangements of partners and shared objects. The solution proposed in [67] is based on avatar replacement and gesture re-targeting in accordance with the remote physical space. However, such a solution certainly requires the semantic knowledge of both spaces, as well as algorithms for correct gesture adjustment. Although interesting, this approach acknowledges “sudden jumps of avatars” and confusing non-verbal cues. To ease gesture processing/re-targeting, knowledge of previous user’s movements may be beneficial, and algorithms for movement prediction may be applied. Even so, ensuring the accuracy of the predicted movements and gestures becomes critical, and synchronization and calibration are still mandatory.

### 5.3. Gaze Support

Sharing gaze is also informative and of great importance for perceiving the feeling of co-presence by the participants. It enables users to be aware of other participants’ gaze direction and therefore of what they are focused on. This is currently not possible through traditional 2D conference systems. To enable gaze support, first, dynamic and accurate eye tracking is required, and second, gaze direction estimation and visualization must be performed. Currently, eye tracking is supported in some of the HMDs (Microsoft Hololens 2, Magic Leap 2, Meta Quest Pro, Pimax Reality 12K, and Pico 4 Pro [45]). In [53], a system incorporating bi-directional gaze behavior cues in 360° panorama was developed and satisfactory participant feedback was obtained. However, the authors notice that enabling the sharing of fast eye movements and ensuring synchronous visualizations is limited by the system’s bandwidth capacity.

### 5.4. Emotion Support

Sharing emotions through the user’s avatar’s facial expression is a key factor for improving the feeling of realistic co-presence. Three-dimensional facial data acquired by depth sensors, in the sense of the realistic capturing approach, are noisy and impose requirement of further processing. Similar to body movement prediction, AI approaches may be beneficial for emotion recognition and prediction of facial dynamics, and thus for reducing latency and bandwidth. Predesigned human models, on the other hand, are insufficiently authentic and limited in relation to the expression of different types of emotions. Highly deformable face statistical models would be relevant to the task, but still, AI facial expression recognition techniques are needed to understand real human emotions. Lip motion synchronization will also be required to enable realistic mouth movements and to ensure audio and video speech consistency. However, both photorealistic and statistical approaches require obtaining real human facial expressions in detail. This most important step is often limited when users wear HMDs. Even though HMDs provide the highest levels of immersion, at the same time, they occlude a great part of the face, hampering the acquisition process. Just a few HMDs support facial tracking (Pico 4 Pro, Meta Quest, and Pimax Reality 12K QLED [45]). Thus, some inside capturing needs to be applied [101,102,103,104], or again, relying on AI to restore the missing facial area will be necessary [105,106]. This, of course, adds additional processing, quality issues, level of detail limitations, inclusion of exterior to the HDM sensors, etc. The challenges discussed in Section 5 are synthesized in Table 4.

## 6. Other Challenges

### 6.1. Evaluation (Subjective and Objective)

The improvement of HTC system performance necessarily goes through extensive system evaluation and further optimization. The term QoS, known as “The totality of characteristics of a telecommunications service that bear on its ability to satisfy stated and implied needs of the user of the service” by the ITU definition [63], or “A set of service requirements to be met by the network while transporting a flow” according to the Internet Engineering Task Force (IETF) [107], is often used by network and service providers for assessment purposes (for quantifying system performance). However, even the utilization of a set variety of QoS metrics, such as throughput, latency, jitter, signal-to-noise ratio, bit-error rate, packet loss, etc., is always helpful for estimating the technological state of the system; it is not enough to conclude the overall system performance experienced by the end users. This is because QoS by nature does not consider any human contentment or expectations, or any of the contexts of system/service utilization. The term QoE, on the other hand, defined as “The degree of delight or annoyance of the user of an application or service, resulting from the fulfillment of his or her expectations with respect to the utility and/or enjoyment of the application or service in the light of the users’ personality and current state” [62,63], considers the user’s expectations and subjective perceptions.

Despite this, assessing QoE, especially for highly immersive media services, is a very challenging task due to the tangle of many different factors impacting the overall system performance. According to [60,61], these influencing factors (IFs) may be classified into the following three groups. First is the group of human IFs that is strongly subjective and is represented by the user expectations and expertise, experienced level of immersion, simulator sickness, and human vision and hearing specifics. Next is the group of the system IFs, divided by four subgroups, namely content-related factors, media-related factors, network-related factors, and hardware-related factors. To the subgroup of content-related factors belong spatial audio, spatial depth, and spatial-temporal complexity. To the subgroup of media-related factors, terms such as audio and video characteristics, type of compression, storage and transport, bit rate, resolution, frame rate, audio sample rate, and coding delay may be specified. The third subgroup of network factors, or yet transmission factors, incorporates very important parameters such as latency, jitter, required bandwidth, and packet loss. The last subgroup of hardware-related factors includes more user-hardware-specific factors such as displaying physical characteristics (weight, size, heat dissipation, etc.), display resolution, refresh rate, FoV, decoder performance, tracking abilities, and headphone characteristics. The system IFs may be considered as objectively measurable units that directly or not, in a combination between themselves or independently, influence the user’s perception and feeling of comfort, immersion, and presence. The last specified group is that of the context-related IFs. It includes the physical context, the temporal context, the social context, and the task context. All these factors have additional impact on the perceived QoE by HTC system users. For example, depending on the task context, different system IFs may or may not influence the overall QoE. Even if the technological system’s performance is highly optimized, users may experience degraded QoE because of some other factors, and vice versa. If the user’s subjective needs/senses/expectations are optimally fulfilled, the experienced QoE may be excellent, even when the technical system performance is at some point degraded.

The complex combination of the different subjective (human IFs), objective (system IFs), and contextual (the context-related IFs) factors makes the process of QoE assessments extremely challenging. Therefore, the system evaluation must include a very large sample of user participants, being preliminarily trained or not, and performing different tasks in variable conditions. New evaluation metrics, compatible with the 3D data format, must be applied [108]. Meanwhile, along with all technical parameters that need to be evaluated, many different subjective aspects provided by the system must also be considered in the assessment protocol, such as the appearance of realism of avatars or/and environments, behavioral realism, feeling of presence and immersion, system usability, sickness, ease of use, experienced time for task performance, rate of successfully performed tasks, etc. All this additional information should be acquired, analyzed, and evaluated. This is currently conducted by surveys, questionnaires, and interviews, as different rating scales are utilized in terms of quantifying system performance. Machine learning (ML) and AI gain much interest in the task of mapping the experienced subjective sense modalities with the system factors. This process is currently in a development stage, and some new ML/AI QoE assessing models are to be defined in the future [59,61].

Most of the studies examined in this work conduct some basic level of HTC system assessment. However, significantly more work is required in the direction of finding the links between technical system performance and end-user satisfaction, as well as in the direction of users and task specifics.

### 6.2. Security, Privacy, and Ethics

Although really challenging, the problems mentioned above receive great attention among the research community. Security and privacy issues, on the other hand, are not that deeply researched in the context of HTC [56]. To the best of our knowledge, none of the papers describing HTC systems examined in this study seriously consider the need for enhancing security and user privacy. However, the new dimensions of holographic communication reveal new challenges in terms of security and privacy. Taking into account that the holographic data are spatial by nature, this means that they contain much more information than traditional 2D data, e.g., size, shape, surface texture, object placement and orientation, etc. Additionally, when different sensing devices, such as depth cameras, HMDs, haptic gloves, or body suits, are used, user sensitive information may be maliciously acquired, e.g., some biometric data such as face shape, iris or retina data, hand geometry, and fingerprints, or some behavioral data such as specific facial expressions, body movements, or fine-grained user movements [51]. Therefore, these data, needed or not for the purposes of communication tasks, must be reliably secured.

Furthermore, when speaking in the context of different application scenarios, some other types of personal-specific data may also be exploited, so privacy leakages must be avoided. An appropriate example is medical applications such as telemonitoring, teleconsultation, or telesurgery, where personal patients’ data must be protected during processing, transmission, and storage.

In [15,56], the authors indicate the existent security and privacy approaches in MR and related technologies, classified into the following groups: input protection, data protection, output protection, user interaction protection, and device protection. In [57], security challenges in collaborative MR (CMR) systems are discussed, mainly focusing on CMR attack understanding, CMR attack use-case studies, and defense strategies. However, improving security must be performed in the network layer as well. Therefore, secure network architectures, protocols, and mechanisms are strongly required to defend the user-vulnerable data in immersive HTC systems while dealing with great amounts of information [58].

Finally, ensuring secured communication and keeping private data safe is of great importance for increasing users’ trust in the immersive applications, especially if they have more important purposes than just simple communication. It is all up to personal ethics and morality to abuse or not the opportunities provided by immersive technology. However, it is up to the technology developers not to rely on personal ethics to keep user data secured and defended. The challenges discussed in Section 6 are summarized in Table 5.

## 7. Discussion of Implemented HTC Systems

The points mentioned in the previous sections are further confirmed by the analysis of the HTC system implementations for remote communication and collaboration shown in Table 6 and Table 7. These systems are presented in terms of utilized input and output techniques, networking technology, system scalability, avatar embodiment, availability of non-verbal communication cues (gestures, eye gaze, and facial expressions), type of system evaluation, and the insurance of user security and privacy. It is noticeable that various capturing devices are used, but still, they support data acquisition for just visual and partially audible human perception. The other human senses are greatly neglected. Accordingly, output data technologies are also focused on visual content representation. HMDs are mostly utilized, which certainly proves that, thus far, other displaying technologies are neither immersive enough, nor preferred by HTC developers. Spatial audio is rarely performed. Therefore, it may be concluded that the idea of multi-sensory interaction is far from being realized. In reference to the networking part, the research papers that describe how this was performed use different technologies such as MPEG DASH, RTSP, WebRTC, RabbitMQ, and PUN. A unified networking protocol optimized for holographic data transmission does not exist, which forces the developers to be inventive and to rely on technologies that originally have different purposes (WebRTC, RabbitMQ, and PUN). HTC system scalability is considered in many of the examined works. However, it is limited up to a few users at the most, which is much fewer than what traditional 2D conference platforms propose. Regarding avatar embodiment, the highly realistic human representations are the most desired ones, but unfortunately they are not implemented in all systems. Non-verbal communication cues are enabled in many of the discussed works—gestures are the most common (about 50% of the works). However, eye gaze and facial expression applications are limited. System evaluation is implemented in almost all the studies, which is indicative of an ambition to improve system performance. However, the evaluation by user studies must be conducted by observing much larger groups of participants and by examining greater volumes of HTC system aspects. According to the latency assessment provided, the achievement of ultra-low-latency holographic communication is still challenging. We believe that the term “real-time” is no longer appropriate, as different HTC applications may depend on different latency values. Therefore, evaluation should be application-specific and task-oriented, and the assessment must be performed not only by the system developers but also by the experts in the respective field. Finally, as far as we know, none of the examined systems consider any security or privacy strategies.

Based on the reviewed HTC system challenges, and on the above analysis, we can conclude the following: the limitations indicated in [5] are still relevant today, despite advances in I/O technologies, processing power, and the era of 5G. This all make us believe that improving technology alone is not nearly enough to improve HTC systems’ performance. A smart approach must be considered because, as technology improves, there is an increase in the amount of data, user expectations, applications entrusted responsibilities, and malicious attacks. We believe that the smart implementation and utilization of advanced, innovative, and intelligent technological solutions will be the key for opening the world to the realistic holographic experience.

## 8. Future Direction—A Framework for a New Intelligent and Capable HTC System

The above HTC system analysis and listed challenges revealed that the current technology is still not matured enough to face the ever-increasing demands of HTC. Accordingly, we believe that the enablement of HTC applications should not solely rely on technology advancements but also on applying smart ways of HTC system implementation. We anticipate that some compensation of technology limitations can be achieved by benefiting the available CV and AI algorithms. Therefore, an intelligent and capable HTC system is such a system that incorporates the recent technology advancements together with the appropriate AI and CV processing in a reasonable time and cost for HTC system requirements to be optimally fulfilled.

**Figure 5:**
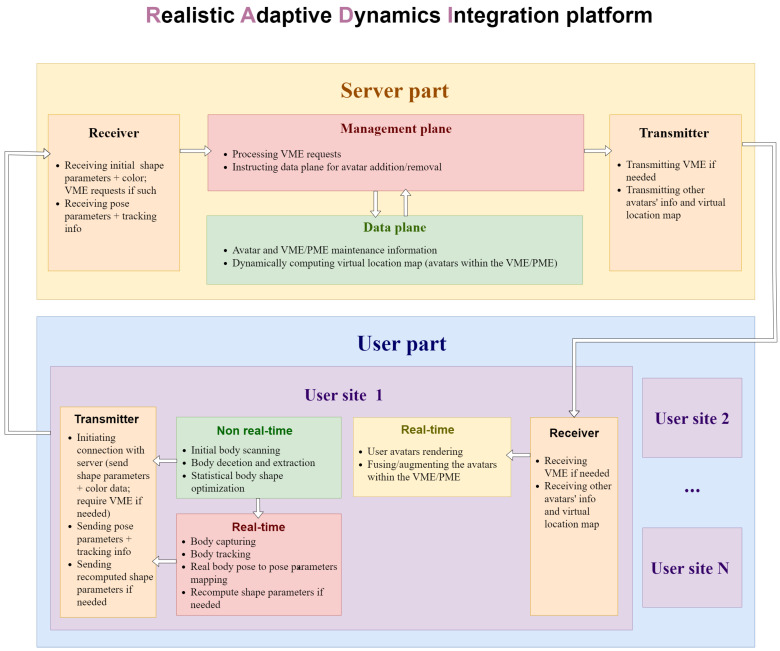
RADI platform.

In this sense, we propose our vision for the development of an immersive holographic communication system. There are a few main principles which are to be pursued during its implementation, namely lightweight processing, ultra-low-latency transmission, ease of scalability, and a high-level of realism in appearance and dynamics. We propose the realization of an HTC system to be based on RADI, which is short for a platform enabled with high-quality “realistic” avatar reconstruction, “adaptive” to arbitrary number of users, and with high-fidelity human body to avatar “dynamics” and “integration”. Assuming the scenario where a random number of users wish to meet virtually in a common 3D space, we propose the following RADI platform architecture, presented in Figure 5.

The framework consists of two main functional units—a server part (for a centralized control and data management) and a user part (representing all user sites participating in the communication). The common 3D space may physically exist within one (or more than one) of the user sites’ physical spaces. For better understanding, we call it a physical meeting environment (PME). It will be populated with remote users’ avatars during the communication. However, the same space may be virtually obtained by the users if they do not physically occupy it. Thus, a virtual twin of the PME must be created and synchronously maintained by the server part. We call this virtual twin space a virtual meeting environment (VME). At each user site, spatial data acquisition, spatial data reproduction, and intended processing are performed. In turn, the server part is responsible for both managing the communication between the users and for the data fusion and augmentation within the VME and PME, respectively. It is important to note that the information transferred between the platform entities is significantly reduced by utilizing innovative compression techniques cooperatively exploiting the potential of AI and statistical body models [111,112]. A more detailed description of the two main functional units and for the operations they are responsible for is given in the following subsections.

### 8.1. User Site

As already mentioned, the “user part” is the same for all the users (user sites) that participate in the communication. In this section, we describe the operations performed by a single user site.

First, the user is captured by at least three depth sensors that are previously calibrated and synchronized. The range of the user’s locomotion is limited only by the range of the cameras used, so his/her body is entirely captured from all sides. Second, the body is detected and extracted from the rest of the acquired scene. A semantic knowledge for human bodies (and their surrounding environment) is essential for speeding up and increasing the accuracy of the body detection and extraction. Third, statistical body model shape optimization is performed according to the real body acquired data. Possible optimization approaches are the ones that utilize Gauss–Newton, Powell’s dog leg, and Levenberg–Marquardt optimizations, respectively, where the statistical model to the real data fitting is defined as a non-linear least-squares problem. The shape of the statistical body model is characterized by its shape parameters, which are pliable to alternation during optimization. It is important to note that executing such a procedure is a computationally expensive task. However, body shape optimization may be performed only once before the communication begins. Hence, it will be responsible for obtaining just the altered statistical body shape parameters. Then, the modified statistical body will be representative for the real body shape. Note that each user site, as well as the server, will be provided with the statistical model, so just transferring shape parameters would be enough to replicate the real human shape everywhere within the platform. Thereby, significant reduction in the transferred 3D data may be achieved. Additional color and texture data are, however, required to create a complete user avatar. Next, avatar movements must reflect the real body dynamics. The pose of the statistical body (i.e., the pose of the already modified by shape statistical body model or the avatar) is defined by the statistical pose parameters, which are also pliable to alternation and must accurately map to the real body pose. For the mapping process, the power of AI modeling and prediction with past frame information is beneficial. However, the AI decision making must be very lightweight and performed in a low-latency manner. Therefore, a preliminary data set of human dynamics must be collected, and the AI model must be trained while movement prediction is also considered. Note that as well as the original statistical body model, the trained AI model will be provided to the rest of the user sites. Thus, the estimation of the pose parameters will completely define all users’ avatar dynamics, and the main informational flow between RADI platform entities will be the shape parameters (just at the beginning) and the pose parameters (during the communication). However, additional shape improvements are not excluded but this time at better initial conditions.

### 8.2. Server Site

The server part is responsible for managing the communication between users, for accurate data fusion within the VME, and for accurate avatar augmentation in the PME. Therefore, two logical planes can be distinguished. The first is the management plane, which maintains the connections between the user sites. Each time a user initiates a connection, the server obtains avatar shape data and possibly a request whether to provide VME. Then, the server must pass the required information and other user sites’ avatar data to the user. Meanwhile, the other user sites must also be updated with the information for the new user. The functions of the data plane are to maintain and update the VME and to support a dynamic virtual map of all the users’ avatar locations and movements within the VME and the PME. If needed, this map will be explicitly adjusted to avoid different avatars colliding with each other or with the obstacles in the VME or the PME, such as walls, chairs, tables, etc.

## 9. Conclusions

HTC has the potential to connect people without making them travel to physically meet each other. The reviewed HTC systems clearly prove there are great efforts on the topic which are made by different groups of researchers.

In this paper, we first propose the architecture of a basic HTC system and the processes that it should perform, reveal the main challenges to the implementation of such systems, and categorize them in three majors groups—main technological challenges (related to I/O technology, data processing, data transmission, and system scalability), representation challenges (namely avatar embodiment, gesture, and gaze and emotions support), and other challenges (more specifically, HTC system evaluation and security and privacy). Furthermore, we extend our review by comparing a number of HTC systems according to the above-mentioned challenges. However, there are some HTC system limitations that are not addressed in this review. These are the challenges related to some more specific data processing such as real-time user/object detection, real-time user/object tracking, users’ avatar/object positioning and gesture re-targeting, if such is required (in order to avoid avatar-to-avatar or avatar-to-environment collisions), mesh reconstruction, avatar body and face rendering, movements, gestures, and face expression predictions. Since the applied processing depends on the concrete application, it may include a variety of the mentioned operations. Other challenges, which we missed addressing, are users trust and acceptance of this type of technology and how they will result in the further evolution of HTC. How will HTC reflect user psychology is also not discussed in the current review, but it is, however, quite important considering the preservation of HTC users’ mental health. Nevertheless, based on the analysis of the state of the art, we conclude that the limitations indicated in [5] are still relevant today. This make us believe that technology improvements themselves are not enough to fully enable HTC, since user data and user expectations increase as well.

Therefore, we proposed a concept for a future HTC system realization, where the smart utilization of technologies is expected to achieve the desired lightweight processing and low-latency transmission with the support of an arbitrary number of users.

## Figures and Tables

**Table 4:** Representation challenges.

Group of Challenges	Challenges
**Avatar Embodiment**	High sense of presence and realism, detailed avatar faces and facial expressions, completeness of avatar bodies and their position, fidelity of movements and gestures, locomotion synchronization between the avatar and the user, sense of body ownership and recognizing users’ self-movements, and avatar placement and cooperation in the interaction space [51];
	Need for higher visual and behavioral avatar fidelity [24,47,49,50,54,66,67,68,77,96,99,100].
**Gestures Support**	Dynamic and accurate body tracking and replicating gestures and body movements by virtual avatars (human to avatar synchronization) [67];
	Calibration and synchronization between different users’ coordinate systems.
**Gazes support**	Dynamic and accurate eye tracking;
	Gaze direction estimation and visualization [53].
**Emotions support**	Dynamic face capturing/tracking and lip to voice synchronization;
	HMD face occlusions [101,102,103,104,105,106].

**Table 5:** Other challenges.

Group of Challenges	Challenges
**Evaluation**	QoS—not enough to assess overall system performance experienced by the end users;
	OoE—tangle of many different factors impacting the overall system performance [60,61];
	Evaluation by a very large sample of user participants, preliminarily trained or not, and performing different tasks in variable conditions;
	Evaluation of variable subjective sense modalities as appearance realism of avatars or/and environments, behavioral realism, feeling of presence and immersion, system usability, ease of use, experienced time for task performance, rate of successfully performed tasks, etc;
	New evaluation metrics compatible with the 3D data format [108];
	Developing ML/AI QoE assessment models [59,61].
**Security and Privacy**	Neglecting the enhancement of security and privacy in HTC systems [56];
	Need for securing more informative 3D data and representing human bodies and environments (size, shape, surface texture, object placement and orientation, etc);
	Need for securing acquired user sensitive data (biometric data such as face shape, iris or retina data, hand geometry, fingerprints, or behavior data such as specific facial expressions, body movements, or fine-grained user movements [51]);
	Need for securing application-specific data from privacy leakage;
	Improving security at the network level while dealing with a great amount of data [58];
	Increasing user trust in immersive applications, especially for purposes different than just plain communication.

**Table 6:** HTC systems reviewed according to input and output technology, and networking.

Reference	Input	Networking	Output
[47]	Xsens suits, HTC Vive with hand controllers	PUN	A tablet device, ODG R7 smart glasses, and HTC Vive
[49]	Microsoft Azure Kinect, Microsoft HoloLens 2 eye and hand tracking, SALSA LipSync, HTC VIVE Pro Eye camera, HTC lipsync facial tracker, and HTC VIVE trackers	RTSP	HTC Vive Pro Eye HMD, and Microsoft HoloLens 2
[50]	HTC Vive head tracking, Vive Controllers, 4 OptiTrack Prime 13 cameras, and Microsoft Kinect V2	Unity Multiplayer and Networking API	HTC Vive and CAVE display
[52]	Leap Motion, aGlass gaze tracking, and Cameras	Not discussed	HTC HMD, aGlass, and Projector
[53]	HoloLens 2 eye tracking, 360° Ricoh Theta camera, and Vive Pro Eye VR HMD	Not discussed	VR HMD
[64]	360° Ricoh Theta V, Samsung 360° Round, Insta360° Pro 2, HMDtracking, and Oculus Touch controllers	LAN using UDP with 1 Gbps Cat6 Ethernet cable	Oculus Rift CV1, video see-through HMD (Vive Pro) by attaching a ZED Mini camera to the front of the HMD
[66]	Stereo cameras, Monochrome tracking cameras, RGBD capture pods, and microphone array	WebRTC	Head-tracked autostereoscopic display
[67]	RGB-D ZED mini camera, Vive Pro HMD tracking, Vive trackers, and Hi5 VR Gloves	PUN	HTC Vive Pro headset
[68]	Microsoft Kinect v2	RabbitMQ	VR HMD
[70]	CAD model processing and scene creation	FFmpeg library for video transmission	Tablets
[71]	RGB-D cameras, Immersive trackable HMD	Not discussed	Immersive trackable HMD
[72]	Depth sensor and microphone array	Not discussed	HTC Vive, large TV display
[76]	Microsoft Azure Kinect, microphones, and ZED stereo cameras	Gbit Ethernet LAN, WebRTC	Looking Glass LFD
[77]	Microsoft Azure Kinect, HMD eye tracking, and Vive trackers	RTSP	Microsoft Hololens 2 and HTC Vive Pro Eye
[78]	Microsoft Azure Kinect	Either DASH or socket-based connections managed by an orchestrator	HMD Oculus Quest 2
[79]	Ricoh Theta-V 360° camera	4G or 5G connection, RTSP	Samsung VR HMD, Samsung Galaxy S8 smartphone
[80]	Intel RealSense D415, VR HMD eye tracking, and Leap Motion	10 Gb Ethernet connection with Draco5 library	HTC Vive Pro Eye and Magic Leap One
[81]	Microsoft Kinect	WAN through 100 Gbps optical links	Oculus Rift
[84]	Intel Realsense D415 cameras and Intel Realsense D435	MPEG DASH	Oculus Rift
[85]	Microsoft Azure Kinect, Intel RealSence cameras, an OptiTrack optical marker system, IMU-equipped gloves, and wireless pen	Network Library for Unity implementing UDP and TCP transmission, WebRTC	Microsoft HoloLens v1 and HTC Vive Pro
[86]	Intel RealSense, Microsoft Azure Kinect	WebRTC	Browser-based platform
[87]	Microsoft Kinect v2, Microsoft Azure Kinect, and Intel RealSense	WebRTC	VR headset
[88]	Apple iPhone 12 Pro, Hand trackers	PUN	Microsoft HoloLens 2 and Oculus Quest 2
[89]	Intel RealSense D435, Intel RealSense T265, Leap Motion, and Oculus Touch device	PUN	Project NorthStar HMD and Oculus Rift
[90]	HTC Vive head tracking, Leap Motion, and Huawei smartphone	Photon Networking Engine	HTC Vive and Huawei smartphone
[94]	Intel RealSense Camera, Leap Motion, VR Controllers (Vive, Oculus), and Motion Trackers (Ptitrack and Kinect)	Not discussed	VR HMD and AR tablet
[95]	MR 360° camera, HTC Vive controller	Not discussed	Large semi-encompassing immersive display
[96]	AR, VR headset tracking, Vive controllers, and Microsoft HoloLens hand tracking	Not discussed	Microsoft HoloLens, HTC Vive
[97]	RGB-D sensors in mobile phones and Microsoft Kinect	Not discussed	VR HMD
[98]	Touch controllers	UDP network	Oculus Rift CV1
[99]	360° camera, Windows MR handheld motion controllers, and HP Windows MR/VR headset sensors	not discussed	Microsoft HoloLens and HP Windows MR VR headset
[100]	HTC Vive Pro head tracking and Vive controllers	1 GBit Ethernet connection	HTC Vive Pro
[109]	Logitech Web camera and Leap Motion	Unity 3D, WampServer, and Microsoft’s Mixed Reality Toolkit	Microsoft HoloLens and HTC Vive Eye Pro Kit
[110]	Zed mini depth camera	not discussed	Samsung Odyssey+

**Table 7:** HTC systems reviewed according to system scalability, avatar embodiment and non-verbal cues, system evaluation, and security and privacy concerns.

Reference	Scalability	Avatar Embodiment	Non-Verbal Cues	User Study	Latency Evaluation	Security and Privacy
[47]	✓	✓	✗	✓	✗	✗
[49]	✗	✓ (both type of avatars)	✓ (limited finer gestures and gaze)	✓	✓ (566.30 ms for predesigned avatars, and 502.31 ms for photorealistic avatars)	✗
[50]	✓	✓ (both type of avatars)	✓ (gestures)	✓	✗	✗
[52]	✗	✗	✓ (gestures and gaze)	✓	✗	✓
[53]	✗	✓ (pre-designed avatars)	✓ (eye gaze)	✓	✗	✗
[64]	✗	✓ (pre-designed avatar)	✓ (gesture and gaze)	✓	✓ (1.2 s)	✗
[66]	✗	✓ (realistic avatar)	✓ (eye contact, hand gestures, and body language)	✓	✓ (off-to-on transition of an LED (105.8 ms))	✗
[67]	✗	✓ (pre-designed avatar)	✓ (gestures and pose)	✓	✗	✗
[68]	✗	✓ (realistic avatar)	✗	✓	✗	✗
[70]	✓	✗	✗	✓	✗	✓
[71]	✓	✓ (realistic avatar)	✗	✗	✓ (0.5 ms)	✗
[72]	✗	✓ (pre-designed avatar)	✓ (database-based facial expressions, gestures, and gaze)	✓	✗	✗
[76]	✗	✓	✗	✗	✓ (210–230 ms for the 29–30 fps in duplex mode)	✗
[77]	✓	✓ (both types of avatars)	✓ (animating eye movements)	✗	✓ (300–400 ms)	✗
[78]	✓ (yes—up to 6 users)	✓ (realistic avatar)	✗	✓	✓ (180.5 ms and 251.2 ms for 2 and 4 point clouds)	✗
[79]	✗	✗	✓ (gaze)	✓	✓ (2K, 6 Mbps on 400 ms, 4K for about 1 s)	✗
[80]	✗	✗ (pre-designed avatar)	✓ (gaze and gestures and head position)	✓	✓ (about 300 ms and fewer than 10 ms for the cues)	✗
[81]	✗	✓ (realistic avatar)	✓ (gestures)	✓	✓	✗
[84]	not discussed	✓ (realistic avatar)	✗	✗	✓	✗
[85]	✗	✓ (pre-designed avatar)	✓ (gestures)	✓	✗	✗
[86]	✓	✓ (realistic avatars)	✗	✗	✗	✗
[87]	✓	✓ (realistic avatar)	✗	✗	✓ (depth transmission evaluation)	✗
[88]	✓	✓ (pre-designed avatar)	✓ (head and hand poses)	✗	✗	✗
[89]	✗	✓ (realistic avatar)	✓(gestures)	✓	✓	✓
[90]	✓	✓ (pre-designed avatar)	✓ (gestures)	✓	✗	✗
[94]	✓	✗	✗	✓	✓	✗
[95]	✓	✓ (realistic avatar)	✓ (gestures, facial expressions, and body language)	✓	✗	✗
[96]	✓	✓ (pre-designed avatar)	✓ (gestures)	✓	✗	✗
[97]	✓	✗	✗	✓	✓	✗
[98]	✓	✓ (pre-designed avatar)	not discussed	✓	✓ (about 10 ms)	✗
[99]	✗	✓ (pre-designed avatar)	✓ (gestures and head gaze, mouth flapping, and periodically blinking)	✗	✗	✗
[100]	✗	✓ (pre-designed avatar)	✓ (gestures and head gaze)	✓	✗	✗
[109]	✗	✗	✓ (gestures)	✓	✗	✗
[110]	✗	✓ (pre-designed avatars)	✗	✓	✗	✗

## Data Availability

Not applicable.

## Abbreviations

The following abbreviations are used in this manuscript:

2D	Two-dimensional
3D	Three-dimensional
5G	Fifth-generation mobile network
AI	Artificial intelligence
AR	Augmented reality
CMR	Collaborative mixed reality
DASH	Dynamic adaptive streaming over HTTP
FOV	Field of view
HMD	Head-mounted display
HTC	Holographic-type communications
I/O	Input/output
IETF	Internet Engineering Task Force
IF	Influencing factor
ITU	International Telecommunication Union
ML	Machine learning
MPEG	Moving Picture Experts Group
MR	Mixed reality
PCC	Point-cloud compression
PME	Physical meeting environment
PUN	Photon unity network
QoE	Quality of experience
QoS	Quality of service
QUIC	Quick UDP Internet connections
RADI	Realistic adaptive dynamic integration
RTSP	Real-time streaming protocol
TCP	Transmission control protocol
ToF	Time of flight
UDP	User datagram protocol
VME	Virtual meeting environment
VR	Virtual reality
WebRTC	Web Real-time communication
XR	Extended reality
